# Bilateral abducens nerve palsies and urinary retention caused by the rupture of a vertebral artery aneurysm: A case report and literature review: Erratum

**DOI:** 10.1097/MD.0000000000010037

**Published:** 2018-02-23

In the article, “Bilateral abducens nerve palsies and urinary retention caused by the rupture of a vertebral artery aneurysm: A case report and literature review”,^[1]^ which appeared in Volume 97, Issue 3 of *Medicine,* the authors would like the add this acknowledgement:

The authors wish to acknowledge Dr. Kang Wang (Department of Neurology, First Affiliated Hospital, Zhejiang University School of Medicine, China) for his assistance in the patient’ diagnosis. We also thank Dr. Jian-wei Pan and Dr. Jie-sheng Zheng (Department of Neurosurgery, Zhejiang University School of Medicine, China) for their assistance in the patient’ continued treatment.

## References

[1] Peng G Zhou J Bilateral abducens nerve palsies and urinary retention caused by the rupture of a vertebral artery aneurysm: A case report and literature review. *Medicine*. 2018 97:e9155. 29504967 10.1097/MD.0000000000009155 PMC5779736

